# A First Approach for the In Vitro Cultivation, Storage, and DNA Barcoding of the Endangered Endemic Species *Euonymus koopmannii*

**DOI:** 10.3390/plants13162174

**Published:** 2024-08-06

**Authors:** Balnur Kali, Sara Bekkuzhina, Dilnur Tussipkan, Shuga Manabayeva

**Affiliations:** 1 Plant Genetic Engineering Laboratory, National Center for Biotechnology, Astana 010000, Kazakhstan; kali@biocenter.kz (B.K.); sara-bek@yandex.ru (S.B.); tdilnur@mail.ru (D.T.); 2 Faculty of Natural Sciences, L.N. Gumilyov Eurasian National University, Astana 010000, Kazakhstan

**Keywords:** *Euonymus koopmannii*, direct regeneration, micropropagation, slow growth storage, DNA-barcoding

## Abstract

*Euonymus koopmannii* is a rare and protected species in Kazakhstan, valued for its ecological role in soil stabilization and its ornamental properties. This study presents the first use of micropropagation and phylogenetic analysis for the endemic plant *E. koopmannii*. Seedlings of *E. koopmannii* proved to be more effective than internodes as primary explants for plant micropropagation of in vitro culture, with a multiplication coefficient of 28.5 from seedlings and 6.1 from internodes. On MSR I medium supplemented with 0.5 mg/L IBA and 0.05 mg/L IAA, a higher success rate of 67% was achieved for root formation of test tube-grown *E. koopmannii* plants. Using mannitol as an osmotic agent at a concentration of 8 mg/L prolonged the storage time of *E. koopmannii* under slow growth conditions when compared to CCC and abscisic acid. Phylogenetic relationships and species identification were analyzed using four DNA-barcoding markers, comparing *E. koopmannii* with species from NCBI. All candidate barcoding markers showed sufficient levels of interspecific genetic variation among *Euonymus* species. In addition, ITS region and *rbcL* gene sequences effectively distinguished *E. koopmannii* from other species. These results provide fundamental information that will be valuable for future biotechnological and molecular studies.

## 1. Introduction

*Euonymus* L. of the family Celastraceae and genus *Euonymus* includes 220 species [1] of shrubs, woody climbers, and small trees, both deciduous and perennial. They are primarily native to East Asia (about ninety-five species), extending to the Himalayas, and are also found in Southeast Asia (twelve species), Europe (four species), Australasia (two species), America (seven species), and Madagascar [2]. *Euonymus koopmannii* Lauche, also known as *Euonymus nanus* var. *turkestanicus*, is a species first described in *Flora Taurico-Caucasica* (1819) and later in Gart.-Zeitung (1883) [3]. *E. koopmannii* is native to Kazakhstan and grows in the Sary-Chelek and Aksu-Zhabagly reserves. It is listed as a rare protected relict species in the Red Book of Kazakhstan [4], although it is classified as Least Concern in the 2007 IUCN assessment (https://www.iucnredlist.org; accessed on 19 February 2024). *E. koopmannii* grows on shady, rocky mountain slopes, in forests, river valleys, and among bushes. This shrub, ranging from 2 to 3 m in height, features underground stems, upright shoots, thin polygonal branches, and fleshy leaves of various shapes. The solitary or clustered flowers bloom in June and pear- or heart-shaped fruits ripen to pink in July and August. *E. koopmannii* is propagated by seed and vegetatively. It plays an important role in soil stabilization and is valued for its ornamental properties [5,6]. *Euonymus* species have been traditionally used as a medicinal plant in many Asian countries to treat injuries, inflammation, cancer, diabetes, and hyperglycemia. The phenolic profile of *Euonymus* was investigated using liquid chromatography–mass spectrometry, and 32 compounds with a high flavonoid contents were identified, including quercitin and kaempferol glycosides [7,8]. The efficacy of *E. alatus* extracts as immunostimulatory agents has been evaluated in macrophages, splenocytes, and an immunosuppressed rat model [9], and scientific evidence supports its role as an anticancer agent [10]. However, the large-scale production of pharmaceutical metabolites from *Euonymus* spp. is challenging due to seasonal variations, and these plants are not typically grown in plantations. Zhu et. al. reviewed phytochemical and bioactivity studies on *Euonymus*, highlighting over 230 compounds, such as sesquiterpenoids, diterpenoids, triterpenoids, flavonoids, phenylpropanoids, lignans, steroids, and alkaloids [11].

With up to two-thirds of the world’s plant species being potentially threatened with extinction by the mid-21st century, the establishment of biobanks is critical. Popova E.V. et al. reviewed global biobanking practices, emphasizing in vitro culture methods for the conservation of ornamental, medicinal, and endangered plants [12]. In vitro culture is effective for synthesizing desired compounds as seen in *E. maximoviczianus*, where the triacylglycerol saturation index and anthocyanin content increase in cultured biomass compared to seedling tissues [13]. Establishing an efficient in vitro micropropagation and plant organogenesis protocol is critical to improving the conservation and utilization of plant resources. This method allows for the large-scale production of genetically uniform plants, which facilitates their conservation and micropropagation. It also provides a platform to study and manipulate biochemical and physiological pathways responsible for production of bioactive compounds with potential applications in medicine, agriculture, and industry. Efficient direct and indirect plant regeneration systems have been established for *E. alatus* facilitating mass micropropagation and the production of pharmaceutical metabolites [14,15,16]. In addition, Ning et al. [17] reported a rapid leaf regeneration system for *E. bungeanus*, and Shang et al. achieved successful regeneration of adventitious shoots from hypocotyl explants of *E. japonicus* [18]. However, there is no information on the in vitro regeneration system of *E. koopmannii*.

In vitro, slow growth storage (SGS) is effective in conserving plant biodiversity, especially endemic plants. Although SGS protocols have been optimized for some species, and many require further research, SGS also protects species from quarantine infections, such as the *Xylella fastidiosa* outbreak in Italy [19]. Many plant growth retardants, such as abscisic acid (ABA), chlorocholine chloride (CCC), and mannitol have been found to be effective in extending the storage life of in vitro-grown tissues [20,21].

Recent studies have investigated the genome sequences of several *Euonymus* species including *E. japonicas* [22,23,24], *E. schensianus* [25], *E. hamiltonianus* [23,26], *E. maackii*, *E. fortunei*, *E. phellomanus* [1,27], *E. szechuanensis* [28], *E. microcarpus* [29], *E. europaeus* [23], and *E. alatus* [30]. However, the chloroplast and mitochondrial genomes of *E. koopmannii* from Kazakhstan remain to be sequenced, and there is no biotechnology and molecular research on this species.

*E. koopmannii* requires micropropagation, conservation [31], and species identification using universal markers as a relict plant. DNA barcoding, widely used in biodiversity conservation, uses standardized universal DNA barcodes such as *rbcL*, *matK*, *trnH*-*psbA*, and ITS for plant species identification [32,33,34,35]. This is the first biotechnological and molecular research on *E. koopmannii*. The objectives were (1) to develop a rapid and efficient protocol for the vegetative micropropagation of *E. koopmannii* by in vitro culture, and (2) to determine the phylogenetic position of *E. koopmannii* through phylogenomic analysis using barcode sequences of *Euonymus* species from the NCBI database.

## 2. Results

### 2.1. Explant Sterilization

During the investigation of the sterilizing agents’ effects, it became apparent that not all explants could withstand extended exposure times under sterilizing conditions, as shown by the data presented in Table 1.

The results of these studies indicated that up to 87.5% of sterile shoots can be obtained using seeds as explants in the second stage with 5% sodium hypochlorite and Tween 20 for five minutes. However, it was noted that using 5% sodium hypochlorite and Tween 20 in the second stage of sterilization for 10 min can cause burns and death of axillary buds of *E. koopmannii*. In addition, sterilization with 1% KMnO_4_ solution was inefficient, reducing the yield of sterile shoots to 43.3%.

### 2.2. Direct Shoot Regeneration from Internodes and Nodal Explants

**Figure 1:**
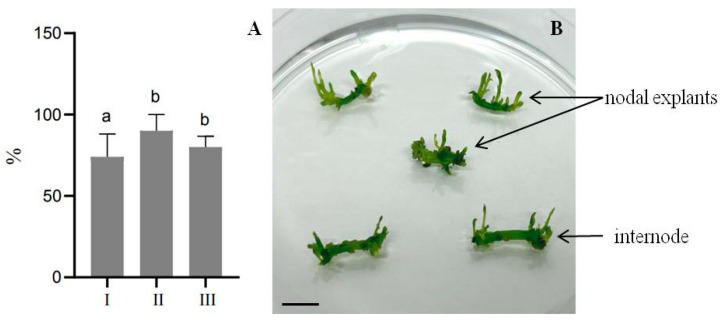
Effect of different concentrations and combinations of cytokinins and auxins on shoot regeneration from *E. koopmannii* in tissue culture. (**A**) Frequency of direct regeneration on MSDR I, II, and III medium; (**B**) Direct shoot regeneration from internodes and nodal explants. Data represent three independent experiments’ mean ± standard deviation (SD) Different letters on the bar indicate statistically significant differences at *p* < 0.05 Tukey’s (HSD), scale bar 1 cm.

A series of experiments on direct shoot regeneration from internodes and nodal explants showed no significant differences in the regeneration potential between different variants of auxin and cytokinin concentrations, as shown in Figure 1. For example, on the MSDR II medium, the regeneration potential reached 90% when plant growth regulators (PGRs) such as 1.0 mg/L BAP, 0.5 mg/L NAA, and 0.2 mg/L kinetin were added, while the regeneration processes did not show any significant variation in the MSDR I and MSDR III media. However, it was observed that these regenerants were unsuitable for micropropagation, which was confirmed in subsequent experiments. Therefore, it was decided to first induce rhizogenesis before proceeding with micropropagation. Primary regenerants with a height of 2–3 cm were transferred to media designed to induce rhizogenesis, using full- and half-strength MS medium supplemented with auxins such as IBA, IAA, and NAA.

### 2.3. Root Formation

**Figure 2:**
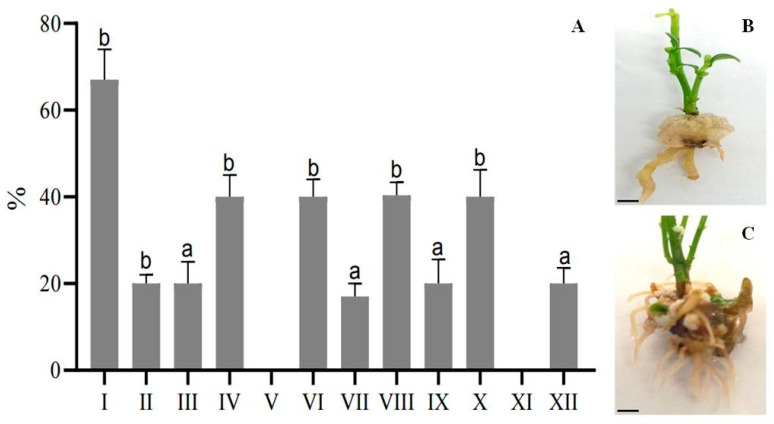
Dynamics of growth and development of *E. koopmannii* roots during in vitro culture. (**A**) Frequency of rhizogenesis of *E. koopmannii* regenerants on MSR I–XII media; (**B**) 8-week-old plants; (**C**) 14-week-old plants. Data represent three independent experiments’ mean ± standard deviation (SD). Different letters on the bar indicate statistically significant differences at *p* < 0.05 Tukey’s (HSD), scale bar 1 cm.

According to the results presented in Figure 2 and Supplementary Table S1, it was clear that during the initial stage of rhizogenesis, calluses were formed from which the roots subsequently developed. The MSR I medium was effective for root formation in *E. koopmannii*, with a success rate of 67% when supplemented with 0.5 g/L IBA and 0.05 mg/L NAA. Conversely, no root formation was observed when adding 2 mg/L IBA and 0.5 mg/L NAA. The cultivation of plants on a nutrient medium containing a full strength of MS salts along with varying concentrations of IBA ranging from 0.5 to 2 mg/L, NAA and ranging from 0.05 to 0.5 mg/L resulted in a root formation rate of 40%. In addition, Figure 2 highlights the morphogenic processes of the plants, particularly the intense root formation observed on a half-strength MS medium.

### 2.4. Micropropagation of Plants from Internode Explants and Seedlings

**Figure 3:**
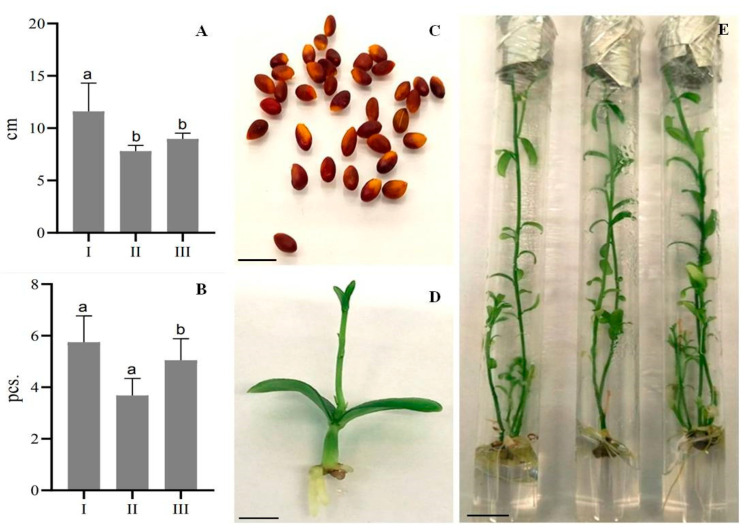
Effect of subculture on elongation of shoot length development and shoot formation of *E. koopmannii* on MSDR I, II, and III medium: (**A**) average shoot length, cm; (**B**) number of shoots per explants, pcs.; (**C**) seeds; (**D**) 4-week-old seedlings; (**E**) 12-week-old plants. Data represent three independent experiments’ mean ± standard deviation (SD). Different letters on the bar indicate statistically significant differences at *p* < 0.05 Tukey’s (HSD), scale bar 1 cm.

Figure 3 presents data on the micropropagation of *E. koopmannii* regenerants induced from seeds (Supplementary Table S2). In the second passage, the microclones showed a significant development delay, with fewer additional shoots than in the first passage. However, in the third passage, an increase in shoot length was observed, up to 9 cm, in contrast to the 7.8 cm length observed in the second passage.

**Figure 4:**
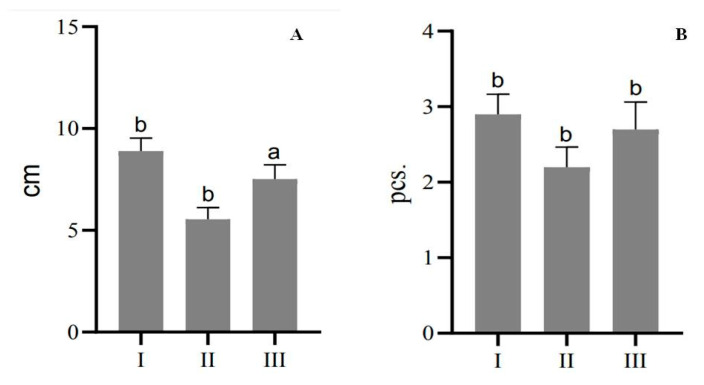
Micropropagation of regenerants obtained from *E. koopmannii* internodes on MSDR I, II, and III medium: (**A**) average shoot length, cm; (**B**) number of shoots per explants, pcs. Data represent three independent experiments’ mean ± standard deviation (SD). Different letters on the bar indicate statistically significant differences at *p* < 0.05 Tukey’s (HSD).

In Figure 3, the number of shoots per explant was higher in the third passage than in the second passage, possibly because the plants were exposed to stress factors after the first passage that initially slowed growth. This phenomenon was evident in two key parameters: average shoot length and number of shoots per explant. After the plant tissues had adapted to the culture conditions, there was a transition from slow growth to intense division and morphogenesis. Micropropagation of *E. koopmannii* from cuttings taken directly from nodal explants proved difficult due to necrosis of more than 90% of newly planted shoots. However, more than five shoots per explant were again obtained when regenerated plants were propagated through the rooting cycle. Notably, when nodal explants of germinated seedlings were used as explants in the micropropagation process of *E. koopmannii,* the plants exhibited intense development, resulting in a higher rate of additional shoot induction compared to regenerants obtained from internodes (Figure 4). Thus, after much experimental work, we increased the number of shoots to three per one explant, including the rooting stages. In addition, longer shoots were obtained in the third passage than in the second passage, where the shoot length decreased significantly compared to the first passage.

The experimental data confirm that an effective method for *E. koopmannii* micropropagation involved plant regeneration followed by rooting and subsequent cutting. Although this method was time consuming, it significantly increased the multiplication rate of *E. koopmannii*. Notably, the fastest and most efficient method for the micropropagation of *E. koopmannii* was the third method described above, which uses germinated seedlings as primary explants (Table 2). The multiplication coefficient of germinated seedlings was 28.5, while that of internodes was 6.1.

### 2.5. In vitro Preservation of Plants by Slow Growth Storage

**Figure 5:**
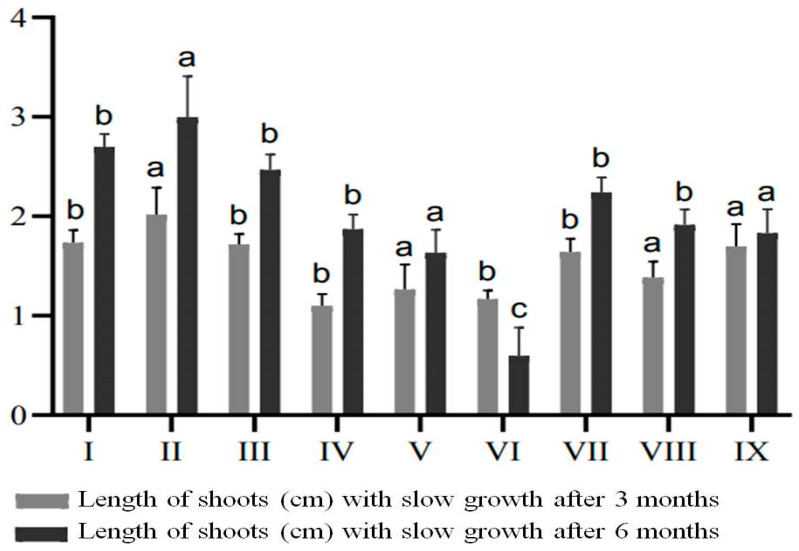
Development of shoot length (cm) during six months of slow growth on MSP I–IX medium. Data represent the mean ± standard deviation (SD) of three independent experiments. Different letters on the bar represent statistically significant differences at *p* < 0.05 Tukey’s (HSD).

For SGS experiments, 157 in vitro micropropagated *E. koopmannii* shoots were used, grown in MSDR II medium supplemented with the hormones 1.0 mg/L BAP, 0.5 mg/L NAA, and 0.2 mg/L kinetin (Supplementary Table S3). The data presented in Figure 5 show the efficacy of growth inhibitors during in vitro storage, evaluated at three and six months. It was observed that the use of ABA, CCC, and mannitol resulted in variations in shoot length in nine variants of media with varying concentrations of retardants. The data showed that, in variants of media with different concentrations of ABA, the length of shoots increased from 0.75 to 0.98 cm with increasing concentrations. Similarly, when the retardant CCC was used, a slight increase in length from 0.19 to 0.6 cm was observed. The addition of ABA and CCC at different concentrations significantly increased plant length. In contrast, when mannitol was added at concentrations of 5, 8, and 10 mg/L, the shoot length was considerably lower compared to the treatments with ABA and CCC.

After six months of cultivation, it was also noteworthy that, when mannitol was added at a concentration of 10 mg/L, a decrease in growth and necrosis was observed, with the shoot length decreasing by 0.57 cm after six months of cultivation. When CCC was used at 1 mg/L, the increase in shoot length was only 0.13 cm, possibly due to the more prolonged exposure to this retardant concentration.

### 2.6. Phylogenetic Analysis

In this study, four barcoding markers were used for the identification of phylogenetic relationships and species. The sequences of *E. koopmannii*, together with 10 *matK* genes, nine *ITS* regions, ten *psbA-trnH* regions, and ten recall gene sequences of *Euonymus* species from the NCBI database, were used to infer the phylogenetic relationships. The length of the sequences was 334 bp for the *matK* gene, 501 bp for the ITS region, 242 bp for the *psbA-trnH* region, and 444 bp for the *rbcL* gene sequences after alignment. The monomorphic sites were arranged at 38–441 bp, and polymorphic sites were 3–139 bp. Genetic variation in the sequences was verified by two neutrality tests, such as nucleotide diversity (Pi) and Tajima’s neutrality test (π). Pi ranged from 0.0012 for the *rbcL* gene to 0.4282 for *psbA-trnH* region 0.19308, while π ranged from 0.0012 for the *rbcL* gene to 0.3833 for the *psbA-trnH* region. A total of 307 bp, 498 bp, 217 bp, and 444 bp nucleotide pairs were analyzed for nucleotide pair frequency analysis in *matK* gene, *ITS* region, *psbA-trnH* region, and *rbcL* gene sequences, respectively (Table 3). The results of nucleotide pair frequency analysis and the maximum likelihood estimation of substitution of the four barcoding regions are shown in Supplementary Tables S4 and S5. The sequences of *matK* gene (accession number OR636392), *rbcL* gene (OR636393), *psbA-trnH* (OR636394), and *ITS* (OR633289) regions of *E. koopmannii* are available in the NCBI database.

**Figure 6:**
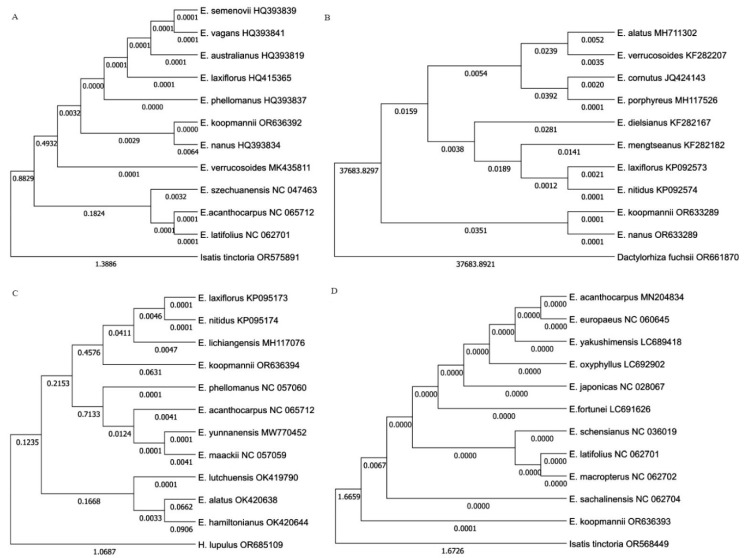
The phylogenetic tree of *E. koopmannii* is based on the maximum likelihood method and the Kimura 2-parameter model. (**A**) *matK* gene. (**B**) ITS region. (**C**) *psbA-trnH* region. (**D**) *rbcl* gene.

The phylogenetic analysis divided *Euonymus* species into two or three main groups based on all candidate barcoding markers (Figure 6). The *matK* gene patterns showed that *E. koopmannii* and *E. nanus* were closely related to *E. semenovii*, *E. vagans*, *E. vagans*, *E. laxiflorus*, *E. phellomanus*, and *E. verrucosoides* with average pairwise distances of 0.00. The *psbA-trnH* region showed that *E. koopmannii* had the same patterns as *E. laxiflorus*, *E. nitidus*, and *E. lichiangensis* with average pairwise distances of 0.06. In contrast, the ITS region and *rbcL* gene sequences of *E. koopmannii* were identified from other *Euonymus* species. Based on the ITS region, sequences *E. koopmannii* and *E. nanus* sequences were grouped in one cluster with average pairwise distances of 0.00 and showed 100% identity. Based on the *rbcl* gene region, only *E. koopmannii* was included in a group with an average pairwise distance of 0.0068 compared to other species.

## 3. Discussion

Micropropagation is widely used in the commercialization of ornamental and fruit crops [36] to conserve disappearing and rare plant species under minimal growth conditions. In addition, micropropagation is essential in the propagation of medicinal plants to select cell lines with maximal production of pharmaceutical metabolites [37]. All plant growth regulators have their own specific effects, and their different combinations cause a specific responce in plants [38,39,40]. This study represents a comprehensive effort to establish the initial stages of tissue culture, define the research strategy for the endemic species *E. koopmannii*, and identify effective types of explants for micropropagation. For example, our results showed that, although the frequency of additional shoot emergence was low when propagating *Euonymus* from stem segments, it was possible to increase the multiplication coefficient by rooting regenerants first and then propagating them again. Protocols for micropropagation and the induction of root-forming potential were developed, demonstrating the versatility of our approach. In addition, the use of germinated *E. koopmannii* seedlings provided sufficient material for further experiments, thus ensuring the sustainability of our research efforts. Direct plant regeneration from stem explants has been successfully achieved in several woody plants such as *Fraxinus pennsylvanica* [41], *Ficus religiosa* L. [42], *Toona ciliata* [43], and *Lycium barbarum* L. [44]. However, studies on the introduction of *Euonymus* species into in vitro culture have not been completed. For example, in the case of *E. bungeanus*, an effective regeneration system was developed using leaf explants induced by 750 nm white light and on half MS medium supplemented with BAP and NAA to form axillary buds [45]. Similarly, the study by A. Shang et. al. showed a high regeneration frequency (63.64%) of *E. japonicus* adventitious shoots induced by culturing hypocotyl segments close to seeds on MS medium supplemented with 1.5 mg/L BAP and 0.05 mg/L NAA [18]. In addition, a highly efficient protocol for shoot regeneration from *E. fortunei* var. radicans stem explants was established, achieving a regeneration frequency of 5.1 shoots per explants [46]. *E. alatus* shoots from axillary buds were rooted in a medium containing 3.0 mg/L IBA [14]. These experiments are consistent with other studies demonstrating the efficacy of half MS medium for root formation. However, in our study we used IAA and IBA at concentrations of 0.05 mg/L and 0.5 mg/L, respectively, instead of NAA. Our results partially confirm previous research, where a similar combination of hormones achieved 77% root formation, albeit with slightly different auxin concentrations than those used in our study [47]. In the species *E. alatus*, the optimization of conditions for the induction of embryogenic suspensions was achieved using the synthetic auxin analog 2,4-D [16]. Based on our research using different types of explants and micropropagation methods, such as direct micropropagation from internodes and nodal segments, it became clear that significant increases in the multiplication coefficient of this endemic plant were achieved only by the process of obtaining regenerates with the root system, followed by their propagation.

Plant genetic resources are conserved using in vitro techniques such as SGS, which regulates plantlet development and reduces handling risks such as contamination and genetic instability. SGS protocols vary based on temperature, light, and media composition, with well-established methods for some fruit species and others requiring further research. Plants respond differently to SGS and micropropagation, being influenced by taxonomic affiliation, growth retardant concentrations, and stress response mechanisms. For example, SGS studies have shown promising results with the addition of CCC at concentrations of 0.2–0.4 mg/L in media for *Chrysanthemum* and *Syringa* L. [48]. Many studies over the last decade have investigated the in vitro conservation of fruit species through the SGS technique and this was reviewed by Benelli et al. in 2022 [20]. The study conducted by Pan et al. found that the most effective method for the slow growth conservation of Vitis heyneana was to use specific media formulations, adjust the osmotic pressure with mannitol (10 g/L), and control environmental factors such as the area of air-breathing film, concentration of CCC (5.0 g/L), and light intensity [49]. Many genotypes of fruit and berry crops were well preserved at 4 °C in bags for cultivation in mannitol (2%), with or without plant growth regulators [50]. When discussing the effect of retardants under slow plant storage conditions in our research, it can be inferred that there is a residual effect from the initial concentrations of mannitol and CCC prior to the six-month storage period. CCC inhibited shoot growth under slow storage conditions, whereas mannitol at 8 mg/L supported optimal growth and did not affect shoot development. Thus, mannitol proved to be the most effective in slowing the height growth of *E. koopmannii*, allowing in vitro storage of shoots for six months at 10 °C with a photoperiod of 16 h of light and 8 h of darkness.

DNA barcoding technology is a powerful tool that uses a short genetic sequence from a standardized genomic region to identify species. This technology has wide applications in various fields, such as species identification, resource management, phylogenetic studies, and evolutionary biology [51,52,53,54,55]. The results of our barcoding study revealed that the average GC content of the ITS region and the *rbcL* gene was higher than the AT content, while the average GC content of the *matk* genes and the *psbA-trnH* region was lower than the AT content. This is a common phenomenon in plants [56]. All candidate barcoding markers satisfied the interspecific genetic variation of *Euonymus* species. However, *E. koopmannii* could not be distinguished from other *Euonymus* species based on the *matK* gene and the *psbA-trnH* region. In contrast, the ITS region and *rbcL* gene sequences were suitable for the molecular identification of *E. koopmannii* at the molecular level from other species. The molecular markers used in this study proved to be valuable in delineating the primary phylogenetic lineages of *Euonymus*. In the comparative analysis using the average pairwise distance, *E. koopmannii* was most similar to *E. nanus*, *E. lichiangensis*, *E. semenovii*, *E. vagans*, *E. laxiflorus*, and *E. phellomanus*. These species belong to the subgenus *Euonymus* section *Euonymus*. On the other hand, *E. koopmannii* was most different from *E. szechuanensis*, *E. cornutus* which belong to subgenus *Kalonymus* and *E. alatus* which belongs to the subgenus *Euonymus*, section Melanocarya. In the research of Yan-Nan LI [2], ITS and ETS markers provided substantial information for understanding the phylogeny of Euonymus, and the molecular patterns strongly supported the idea that subgenera and sections differentiated *Euonymus* species.

In conclusion, our research involves a comprehensive study of an endemic plant, *E. koopmannii*, including its introduction into in vitro culture, the intricacies of micropropagation, storage under in vitro conditions, and determination of temporal growth parameters during non-transplanted culture storage. These results highlight the potential for further research and optimization of tissue culture techniques for *Euonymus* species, including *E. koopmannii*, to contribute to the conservation and sustainable use of these valuable plant resources. The logical continuation of this work was the assignment of a DNA barcode to *E. koopmannii*, which will further enhance our understanding and conservation efforts for this species.

## 4. Materials and Methods

### 4.1. Plant Material and Explant Preparation and Disinfection

All plant materials were collected in the Aksu-Zhabagly Reserve, Maloe Kayindy Gorge (42°24′25″ N, 70°34′49″ E), in the Turkestan region of Kazakhstan, under the guidance of State Reserve botanists. The Forestry and Wildlife Committee of the Ministry of Ecology, Geology and Natural Resources of the Republic of Kazakhstan obtained permission to collect endangered species. Seeds and axillary buds (1.0–1.5 cm) were isolated from the shoots of an actively growing *E. koopmannii,* approximately 30–35 years old and 1.70 m tall, and used as research objects. The experiments were repeated three times independently. In total, 142 axillary buds and 183 seeds of *E. koopmannii* were sterilized in two steps, as detailed in Supplementary Table S6. First, seeds and axillary buds of wild-type *E. koopmannii* were washed in 1.0% sodium hypochlorite and 1% KMnO_4_ solutions for 30 min, followed by thorough rinsing under running water. Then, in the second stage of sterilization, 3% and 5% sodium hypochlorite solutions were used for 5 and 10 min, respectively, with the addition of Tween 20. The explants were then rinsed 3–4 times with sterile distilled water and dried on sterile filter paper before being planted on nutrient media.

### 4.2. Direct Regeneration, Rooting and Micropropagation

Murashige and Skoog (MS) mineral salt-based nutrient media with vitamins (M519, PhytoTech) containing 3% sucrose were used for tissue culture. PGRs were added to the nutrient media for micropropagation (Table 4). The stock solutions of PGRs were prepared individually. For example, 20 mg of auxins were dissolved in a few drops of ethanol, while cytokinins were dissolved in a few drops of 1 N NaOH and made up to 20 mL using distilled water, then stored in the refrigerator at 20 °C. The PGR solutions were sterile filtered through a 0.2 µm filter and added to the chilled media under sterile conditions.

Phytogel at 2 g/L was used as the gelling agent. Axillary buds and seeds of *E. koopmannii* were cultured at 26–28 °C under a 16 h photoperiod, illuminated by fluorescent lamps with an intensity of 2000 lx.

For in vitro rooting of adventitious shoots, auxins such as indolylacetic acid (IAA), naphthylacetic acid (NAA), and indolylbutyric acid (IBA) were used as inducers of rooting processes in different combinations and concentrations as listed in Table 5. The media in the table consisted of half (½) and full-strength MS media supplemented with MS vitamins.

### 4.3. Slow Growth Storage

Half-strength MS medium supplemented with plant growth retardants, such as ABA, CCC, and mannitol was used to regulate the growth of *E. koopmannii* at different concentrations indicated in Table 6. The plants were kept for 6 months under light conditions with an intensity of 1200–1500 lux at a reduced temperature of +10 °C, with a photoperiod of 16 h light and 8 h dark. Observations were made after three and six months when the plants were transferred to standard culture conditions.

### 4.4. DNA Barcoding

For DNA barcoding, young leaves were stored at −80 °C until DNA extraction. DNA was extracted from fresh *E. koopmannii* leaves using the CTAB method [57] with slight modifications. The extracted DNA was checked for its intactness, homogeneity, and purity by 1% agarose gel electrophoresis and run at 120 V for 30 min. The DNA was stored in a freezer (−20 °C) until used in the next step of the experiment. The selection of universal barcode primers was guided by the relevant literature and was fully detailed in Supplementary Table S7. They were synthesized by the Organic Synthesis Laboratory of the National Center for Biotechnology (NCB) (Astana, Kazakhstan). Amplification and sequencing methods were performed according to the standard protocol of the Plant Genetic Engineering Laboratory of NCB [58].

### 4.5. Statistical Analyses

The multiplication rate was calculated according to the following formula:

Multiplication rate = b/a(1)

where

a—number of shoots after the first passage
b—number of shoots after the third passage

For data analysis, the data represent the mean ± standard deviation (SD) of the replicates was analyzed using MS Excel 2021. ANOVA was used to evaluate significant differences, and Tukey’s honestly significant difference (HSD) test (*p* < 0.05) was analyzed using GraphPad Prism 8 software.

Sequence alignment of *Euonymus* species was performed using Vector NTI Advance 11.0 [59] and then used for further analyses. The nucleotide diversity score (Pi) was calculated using DnaSP v5.10 [60]. Phylogenetic analyses were performed using the maximum likelihood method and the Kimura 2-parameter model, and evolutionary divergence between sequences was estimated by the software MEGA 11 [61].

## 5. Conclusions

The present study represents a comprehensive investigation of the endemic plant *E. koopmannii*, including micropropagation, in vitro storage and growth characteristics of *E. koopmannii* under slow growth conditions. The barcoding markers for all candidates corresponded to the interspecific genetic variation of *Euonymus* species. The ITS region and *rbcL* gene sequences were suitable for distinguishing *E. koopmannii* from other species at the genetic level. These results provide fundamental helpful information for future biotechnological and molecular research.

## Figures and Tables

**Table 1:** Effect of sterilizing agents and exposure time on obtaining aseptic culture of *E. koopmannii*.

No of Variants	Sterilization Options				Type of Explant	Shoot Formation In Vitro (%)
	Step 1	Exposure Time	Step 2	Exposure Time		
I	1% sodium hypochlorite	30 min one time	5% sodium hypochlorite + Tween 20	10 min	axillary buds	67.5 ± 0.12
					seeds	63.3 ± 0.10
II				5 min	axillary buds	85.2 ± 0.09
					seeds	87.5 ± 0.07
III	1% KMnO_4_ solution		3% sodium hypochlorite + Tween 20	10 min	axillary buds	49.4 ± 0.14
					seeds	43.3 ± 0.51

**Table 2:** Multiplication coefficient of *E. koopmannii* after three rounds of micropropagation.

Explant Type	Number of Shoots after the First Passage, Pcs	Number of Shoots after the Second Passage, Pcs	Number of Shoots after the Third Passage, Pcs	Multiplication Rate
Seedlings	4	31	114	28.5
Internodes	7	20	43	6.1

**Table 3:** Genetic diversity of *E. koopmannii* based on the candidate barcoding sequences.

	*matK* Gene	ITS Region	*psbA-trnH* Region	*rbcL* Gene
Total aligned length (bp)	334	501	242	444
GC content (%)	32.38	59.6	26.4	45.7
Codon count	101	-	70	148
Number of monomorphic sites	165	403	38	441
Number of polymorphic sites	132	91	139	3
Total number of InDels sites	37	7	65	0
Overlapping InDels sites	0	2	38	0
Number of singleton variables sites	2	41	5	3
Total number of mutations (Eta)	134	107	196	3
Parsimony informative sites (PIC)	130	50	83	0
Nucleotide diversity (Pi)	0.1931	0.0625	0.4282	0.0012
Tajima’s neutrality test (π)	0.1723	0.0494	0.3833	0.0012
Mean nucleotide difference (k)	57.345	30.889	75.782	0.545
Number of Haplotypes (h)	5	9	10	2
Haplotype diversity (Hd)	0.709	0.978	0.982	0.182
Variance of Haplotype diversity	0.01865	0.00292	0.00215	0.02061
Sequence conservation (C)	0.581	0.814	0.199	0.993
Conservation threshold (CT)	0.68	0.91	0.29	1

**Table 4:** Variations of nutrient media for *E. koopmannii*.

PGRs, mg/L	MSDR I	MSDR II	MSDR III
BAP	0.5	1.0	2.0
NAA	0.1	0.5	1.0
Kinetin	−	0.2	−

**Table 5:** Media options for rhizogenesis of regenerated *E. koopmannii* plants.

Options	MS	IBA, mg/L	IAA, mg/L	NAA, mg/L
MSR I	½	0.5	0.05	−
MSR II	full			
MSR III	½	1.0	0.1	−
MSR IV	full			
MSR V	½	2.0	0.5	−
MSR VI	full			
MSR VII	½	0.5	−	0.1
MSR VIII	full			
MSR IX	½	1.0	−	0.5
MSR X	full			
MSR XI	½	2.0	−	0.05
MSR XII	full			

**Table 6:** Variations in MSP medium with retardants for storage of *E. koopmannii* under slow growth conditions.

Variations of MS Medium	ABA, mg/L	Mannitol, mg/L	CCC, mg/L
MSP I	2.0	−	−
MSP II	5.0	−	−
MSP III	10.0	−	−
MSP IV	−	5.0	−
MSP V	−	8.0	−
MSP VI	−	10.0	−
MSP VII	−	−	0.2
MSP VIII	−	−	0.5
MSP IX	−	−	1.0

## Data Availability

All relevant data have been provided as Tables and Figures in the text and Supplementary Materials.

## Supplementary Materials

The following supporting information can be downloaded at: https://www.mdpi.com/article/10.3390/plants13162174/s1, Table S1: Rhizogenesis of *E. koopmannii* regenerants on MSR I–XII media. Table S2: Micropropagation *E. koopmannii* on MSDR II medium containing 1.0 mg/L BAP, 0.5 mg/L NAA and 0.2 mg/L kinetin. Table S3: Slow growing storage data. Table S4: The nucleotide pair frequency analysis of the four bar-coding sequences. Table S5: Maximum likelihood estimate of substitution of the four bar-coding sequences. Table S6: Effect of sterilizing agents and exposure time on obtaining aseptic culture of *E. koopmannii*. Table S7: Nucleotide sequences of PCR primers used for the four bar-coding sequences. References [62,63,64,65] are cited in the supplementary materials.
